# Efficacy of Manual Hemostasis for Percutaneous Axillary Artery Intra-Aortic Balloon Pump Removal

**DOI:** 10.1155/2020/8375878

**Published:** 2020-07-26

**Authors:** Rajiv Tayal, Michael DiVita, Christoph W. Sossou, Alexis K. Okoh, Kelly Stelling, James M. McCabe, Amir Kaki, Najam Wasty, David A. Baran

**Affiliations:** ^1^Division of Cardiology, Newark Beth Israel Medical Center, Newark, NJ, USA; ^2^Division of Cardiology, St. Michael's Medical Center, Newark, NJ, USA; ^3^Department of Internal Medicine, Newark Beth Israel Medical Center, Newark, NJ, USA; ^4^University of Nevada Las Vegas, School of Medicine, Las Vegas, NV, USA; ^5^Advanced Heart Failure Center, Sentara Heart Hospital, Norfolk, VA, USA; ^6^Division of Cardiology, University of Washington Medical Center, Seattle, WA, USA; ^7^Wayne State University School of Medicine, St. John's Ascension, Detroit, MI, USA

## Abstract

**Background:**

The prevalence of peripheral vascular disease has led to the re-emergence of percutaneous axillary vascular access as a suitable alternative access site to femoral artery. We sought to investigate the efficacy and safety of manual hemostasis in the axillary artery.

**Methods:**

Data were collected from a prospective internal registry of patients who had a Maquet® (Rastatt, Germany) Mega 50 cc intra-aortic balloon pumps (IABP) placed in the axillary artery position. They were anticoagulated with weight-based intravenous heparin to maintain an activated partial thromboplastin time (aPTT) of 50–80 seconds. Anticoagulation was discontinued 2 hours prior to the device explantation. Manual compression was used to achieve the hemostasis of the axillary artery. Vascular and bleeding complications attributable to manual hemostasis were classified based on the Valve Academic Research Consortium-2 (VARC-2) and Bleeding Academic Research Consortium-2 (BARC-2) classifications, respectively.

**Results:**

29 of 46 patients (63%) achieved axillary artery homeostasis via manual compression. The median duration of IABP implantation was 12 days (range 1–54 days). Median compression time was 20 minutes (range 5–60 minutes). There were no major vascular or bleeding complications as defined by the VARC-2 and BARC-2 criteria, respectively.

**Conclusion:**

Manual compression of the axillary artery appears to be an effective and safe method for achieving hemostasis. Large prospective randomized control trials may be needed to corroborate these findings.

## 1. Introduction

The femoral artery remains the vascular access site of choice for endovascular procedures requiring large bore arterial access (LBA) including complex coronary intervention, mechanical circulatory support (MCS), endovascular aortic aneurysm repairs (EVAR), and transcatheter aortic valve replacement (TAVR). Both peripheral vascular disease (PVD) and coronary artery disease (CAD) have similar risk factors, and it is common to encounter the challenge of treating structural or complex coronary disease in patients with significant concomitant PAD. Several studies have shown the overall prevalence of PAD in patients undergoing TAVR of about 25%, with outcomes associated with a traditional femoral approach known to be worse in this population [1, 2].

Percutaneous axillary vascular access has recently re-emerged as an alternative percutaneous access option for large bore arteriotomies in patients with severe or occlusive iliofemoral vascular disease and in fact has now become the most utilized alternative access technique in the United States. However, safe axillary vascular access with effective hemostasis requires special techniques which have not been well described in the literature. The efficacy of manual hemostasis for axillary vascular access is unknown. Therefore, we sought to investigate the use and outcomes of manual hemostasis in the axillary artery for the removal of percutaneously inserted intra-aortic balloon pumps (IABPs).

## 2. Methods

Data were collected from a prospective internal registry of patients who had a Maquet® (Rastatt, Germany) 8-French Mega 50 cc IABP placed in the axillary artery position in 46 consecutive patients [3]. A majority of these devices were placed for acute decompensated congestive heart failure deemed to require mechanical circulatory support based on hemodynamic indices and used as a bridge to recovery or destination therapy. The registry included comprehensive data related to the insertion and removal of the device. All the devices were inserted in the cardiac catheterization lab using our previously described techniques for percutaneous axillary access and performed using a combination of palpation, vascular ultrasound, and angiographic visualization [4].

Following device insertion, the patients were most frequently supported with 1 : 1 counter pulsation, and, per our group's standardized strategy, anticoagulated with a 50 U/kg weight-based bolus of heparin and subsequently started on a weight-based drip to maintain an activated partial thromboplastin time (aPTT) of 50–80 seconds for the duration of device of implantation.

Prior to device removal, all patients were assessed for their ability to tolerate weaning of MCS with trials of 1 : 2 and 1 : 3 augmentation. If weaning was tolerated, 1 : 1 augmentation was resumed and anticoagulation discontinued for 2 hours prior to device removal. aPTTs were not routinely assessed prior to the device removal. Patients were brought back to the catheterization lab if their ability to tolerate MCS weaning was uncertain and re-insertion or exchange of MCS device was felt to be a possibility. Otherwise, IABPs were removed at bedside in the Cardiac Critical Care Unit.

We analyzed the methods for achieving hemostasis of the axillary artery at time of IABP removal. Vascular and bleeding complications attributable to manual hemostasis were classified based on the Valve Academic Research Consortium-2 (VARC-2) and Bleeding Academic Research Consortium-2 (BARC-2) classifications, respectively. Additionally, any neurologic complications related to manual compression and hemostasis, mainly brachial plexopathies, were evaluated.

## 3. Results

### 3.1. Study Population

Of the 46 patients who had an IABP placed in the axillary artery, manual compression for hemostasis was used in 29 patients (63%). The remaining patients had the catheter removed with a variety of techniques including closure device (Angio-Seal) or balloon tamponade. No further information is available for these cases. All catheters were placed in the left axillary artery. Baseline characteristics (Table 1) for those patients in whom manual compression was used for hemostasis showed an average age of 55.9 years (range 21–74 years) and a predominance of males (21/29, 72.4). Significant comorbidities included coronary artery disease (3/29, 10.3%), diabetes mellitus (6/29, 20.7%), and chronic kidney disease (11/29, 37.9%). The median LVEF by echocardiography was 19% (range 10–55%). The average BMI was 29.5 kg/m^2^ (range 18–59 kg/m^2^). All patients ambulated, while the IABP was in place (29/29, 100%). The median duration of IABP implantation was 12 days (range 1–54 days). Hemostasis was achieved at bedside in 19/29 patients (65.5%). Median compression time was 20 minutes (range 5–60 minutes).

### 3.2. Removal Technique

In each case, the IABP console was placed on “standby” mode to fully extract helium from the catheter. Next, the skin site of entry was sterilely scrubbed with caution, and then sutures holding the catheter were cut and removed. Manual pressure was exerted medially to the entry site, and the catheter and any sheath were directly removed after initially allowing some bleedback (<10 cc blood). Pressure was exerted while observing the arteriotomy site lateral to the compression to assure no bleeding was noted. Fifteen minutes of occlusive pressure was applied, and then progressive reduction of pressure over 5 minutes until pressure was discontinued.

### 3.3. Safety Outcomes

In the 29 patients in whom manual compression was used to achieve hemostasis of the axillary artery, there were no major vascular or bleeding complications as defined by the VARC-2 and BARC-2 criteria, respectively. Three (3/29, 10.3%) patients experienced a minor vascular complication related to hemostasis, as defined by the VARC-2 classification. Three (3/29, 10.3%) patients experienced a BARC-2 type 1 or type 2 bleeding event. No patients required blood transfusions for bleeding or intervention by vascular surgery. No brachial nerve plexus injuries were experienced (Table 2). Additionally, there were no site-related infections or clinically significant cerebrovascular events following the IABP removal.

## 4. Discussion

Despite the use of newer access sites such as the radial artery or axillary artery, common femoral artery access remains the most commonly used site for access both in the United States and worldwide. This is likely due to the familiarity in obtaining access and achieving hemostasis at this site, which is relatively unchanged since percutaneous access of the femoral artery was first introduced by Sven Seldinger in 1953 [5]. Hemostasis of the femoral artery is predicated on the concept of compressibility against the underlying femoral head. As such, the ideal site for femoral arterial puncture is from the lower border of the head of the femur to the midportion of the femoral head, known as the “target zone” [6]. This site insures an area of compressibility with avoidance of cannulation above the inguinal ligament or below the femoral bifurcation which minimizes complications such as bleeding or hematoma formation.

Axillary artery access is not a new concept. It was originally performed by palpation and cannulation of the artery along the deltopectoral groove or within the true axilla itself, with the arm typically abducted to 90 degrees or positioned above the patient's head. Access was initially performed at this site due to its proximity to the humeral head after drawing on prior experiences seen with traditional percutaneous femoral artery access. Additionally, anatomic studies suggested that abduction of the arm draws the axillary artery over the humeral head, creating an additional point of possible compression much like access in the femoral artery. This was described by Rohrer et al. who analyzed axillary artery thrombosis in baseball pitchers and found that impingement of the third portion of the axillary artery by the head of the humerus occurred in athletes, as well as nonathletes, when the arm was placed in an abducted and externally rotated position, such as would occur with a baseball pitcher's throwing motion [7].

Unfortunately, this technique was found to have an overall complication rate as high as 24%, driven mostly by brachial plexus injury and hematoma formation, which eventually lead to its abandonment [8]. This unacceptably high complication rate associated with early percutaneous transaxillary access performed in this manner was largely attributable to the convergence of multiple brachial plexus elements in the area of access, as well as the presence of the brachial fascial sheath which encompasses the artery, vein, and nerve, wherein even minor bleeding events may lead to a neurovascular compression syndrome.

However, a recent resurgence in interest for percutaneous transaxillary access continues to grow with outcomes on par with traditional percutaneous femoral access aside from a few reports citing an elevated risk of cerebrovascular accident associated with this approach. This has, in large part, been due to data suggesting inferior outcomes with surgical techniques requiring intrathoracic access for transcatheter aortic valve replacements (TAVRs) when transfemoral access is not suitable.

Schäfer et al. were among the first to describe the technique of percutaneous transaxillary artery access for TAVR in a large case series format [9]. As opposed to previous transaxillary access techniques, their technique utilized an anterior approach through the chest wall, entering the axillary artery in its first segment, or in the proximal 1/3 of the vessel. The advantage of this technique includes, among other aspects, potential compressibility of the vessel against the second rib for manual hemostasis, if needed. As such, Schäfer et al. observed a significantly lower complication rate than was reported in the previous studies [10].

In contradistinction to this, our technique for percutaneous transaxillary access has been directed towards an access point in the middle third of the vessel, typically referred to as the second portion, due to a well-defined paucity of brachial plexus elements associated with the artery in this area [11]. Access in this area avoids the risk of entering the intrathoracic cavity which may occur while targeting the first portion of the vessel and can decrease the risk of additional complications, such as hemo-or pneumothoraces, and facilitate surgical cut down and bail out strategies if they were required.

Despite a lack of bony structures in this area to which compression of the artery may be secured against, the findings of our current study, as well as others, suggest manual compression for hemostasis of the axillary artery to be feasible and safe. It is important to note, however, that a lack of bony structures in this area to facilitate manual compression leads to potential displacement of the vessel when attempting compression. As such, attention must be paid when securing hand placement and stabilizing the vessel for manual hemostasis.

In many instances, urgency of device implantation or uncertain duration of implantation deters many physicians from placement of vascular closure devices or from using well described arteriotomy preclosure techniques. This concern becomes further amplified in the cardiac transplantation population as any form of infection or vascular complication could delay the definitive therapy in terms of transplant listing status or potentially increase the risk of infection and, in turn, greater morbidity, given these complex patients require relatively aggressive immunosuppression immediately after transplantation.

More interestingly, our data may suggest significant clinical benefit for the use of percutaneous axillary artery implantation of MCS for prolonged support, given all patients were ambulated and no patients experienced a site-related infection, thus allowing for optimized patient condition prior to transplantation or insertion of durable LVAD. Moreover, no upper extremity ischemia was experienced which may relate to the fact that desired access point in the second portion of the axillary artery is proximal to the subscapularis artery which is known to collateralize the brachial artery.

In our study, all 29 patients had successful hemostasis of the axillary artery achieved with manual compression. Three minor bleeding complications occurred, mainly small chest wall hematomas, which resolved without reintervention or blood transfusion. It should also be noted that one patient utilized manual compression for successful hemostasis after a failed vascular closure device. This suggests that manual hemostasis may offer an alternative to covered stent use as a bailout in certain settings. Furthermore, manual hemostasis could obviate the need for late use of vascular closure devices to achieve hemostasis in patients with prolonged device implantations, thus reducing infection and complication risks.

The first limitation of our study is that patients had axillary artery access performed for the insertion of an intra-aortic balloon pump. This device requires an 8-French (Fr) arteriotomy, whether sheathed or sheathless, and thus not a true “large bore” arteriotomy. This limits the generalizability of this technique to other endovascular procedures utilizing the axillary artery access point. However, our experience with manual hemostasis in the IABP population has led us to develop an increased comfort with utilizing manual compression to facilitate hemostasis in large bore arteriotomies, even those requiring 14–16 Fr arteriotomies. A second limitation is the lack of data available with regards to coagulation testing. All patients were maintained on unfractionated heparin utilizing a standard weight-based dosing while the IABP was in place. Irrespective of aPTT, all IABPs were removed after 2 hours of heparin discontinuation. Fortunately, no major bleeding complications were observed. A third limitation is that, in patients who underwent device removal at bedside, angiography was not utilized to confirm the absence of upper extremity embolization or thrombosis. Thus, it is possible that vascular events could have been greater than observed, although clinically this was not the case. Lastly, in contradistinction to recent literature suggesting higher rates of CVA, we had no events, and this may relate to the size of the device utilized relative to the vessel size, operator experience, or minute differences in procedural technique.

In conclusion, manual compression of the axillary artery appears to be an effective and safe method for achieving hemostasis. Further studies may be needed to corroborate these findings, especially in procedures requiring larger arteriotomies.

## Figures and Tables

**Table 1:** Patient characteristics.

Characteristic	Manual Hemostasis (*N* = 29)
Age–years (range)	55.9 (21–74)
Male–no. (%)	21 (72.4)
Weight–kg (range)	87.5 (40–151.8)
BMI–kg/m^2^ (range)	29.5 (18–59)
Non-African American–no. (%)	26 (89.7)
African American–no. (%)	3 (10.3)
Diabetes mellitus–no. (%)	6 (20.7)
Coronary artery disease–no. (%)	3 (10.3)
Chronic kidney disease–no. (%)	11 (37.9)
LVEF-(%) (range)	19 (10–55)
Access left axillary artery–no. (%)	29 (100)
Ambulated–no. (%)	29 (100)
Median duration of insertion–days (range)	12 (1–54)
Median compression time–min. (Range)	20 (5–60)
Removal at bedside–no. (%)	19 (65.5)

**Table 2:** Safety outcomes.

Outcome	Event (*N* = 29)
VARC-2 major–no. (%)	0 (0)
VARC-2 minor–no. (%)	3 (10.3)
BARC-2 type 3–5–no. (%)	0 (0)
BARC-2 type 1-2–no. (%)	3 (10.3)
Vascular surgery intervention–no. (%)	0 (0)
Bleeding requiring transfusion–no. (%)	0 (0)
Brachial plexus injury–no. (%)	0 (0)
Access site infection–no. (%)	0 (0)
Cerebrovascular accident–no. (%)	0 (0)

## Data Availability

The data supporting the findings of this study are available within the article.
